# QUALITY OF LIFE AND UPPER LIMB FUNCTION OF CHILDREN WITH NEONATAL BRACHIAL PLEXUS PALSY

**DOI:** 10.1590/1984-0462/2020/38/2018304

**Published:** 2020-03-09

**Authors:** Daiane Lazzeri de Medeiros, Natália Borges Agostinho, Luis Mochizuki, Anamaria Siriani de Oliveira

**Affiliations:** a Universidade de São Paulo, Ribeirão Preto, SP, Brazil.

**Keywords:** Child, Quality of life, Upper extremity, Motion, Brachial plexus, Child health, Criança, Qualidade de vida, Extremidade superior, Movimento, Plexo braquial, Saúde da criança

## Abstract

**Objective:**

To compare the upper limb function and quality of life between children with neonatal brachial plexus palsy and controls with unaffected brachial plexus (typical children).

**Methods:**

Twenty-four children with neonatal brachial plexus palsy and 24 typical ones were evaluated, both groups with 10±3 years of age. The upper limb function was assessed by the Modified Mallet Scale and the Active Movement Scale, whereas quality of life was analyzed by the Pediatric Outcome Data Collection Instrument and the Child Health Questionnaire. Mann-Whitney U tests investigated the differences between groups in such scales.

**Results:**

Children with neonatal brachial plexus palsy presented lower limb function compared to typical children in both scales. These children also presented lower scores for most of the Pediatric Outcome Data Collection Instrument domains, except for comfort/pain. In addition, they had lower scores in the following domains of the Child Health Questionnaire: physical functioning, pain, behavior, mental health, overall health perception, emotional impact on parents, and psychosocial summarized score.

**Conclusions:**

Neonatal brachial plexus palsy has a negative influence on upper limb function and quality of life, mainly considering overall health, basic mobility, physical and psychosocial functions, happiness, pain, behavior, mental health, upper limb function, and emotional impact on their parents.

## RESUMO

**Objetivo:**

Comparar a função do membro superior e a qualidade de vida entre crianças com paralisia obstétrica do plexo braquial e aquelas sem paralisia do plexo braquial (crianças usuais).

**Métodos:**

Foram avaliadas 24 crianças com paralisia obstétrica do plexo braquial e 24 crianças usuais, ambos os grupos com 10±3 anos. A função do membro superior foi avaliada pela Escala Mallet Modificada e Active Movement Scale, já a qualidade de vida foi analisada por meio das escalas Pediatric Outcome Data Collection Instrument e Child Health Questionnaire. Foram realizados testes U de Mann-Whitney para investigar diferenças entre os grupos nas escalas.

**Resultados:**

Crianças com paralisia obstétrica do plexo braquial apresentaram menor função do membro superior quando comparadas às crianças usuais, em ambas as escalas utilizadas. Essas crianças também apresentaram menores pontuações para a maioria dos domínios do Pediatric Outcome Data Collection, exceto para conforto/dor. Além disso, apresentaram escores inferiores nos seguintes domínios do Child Health Questionnaire: função física, dor, comportamento, saúde mental, percepção da saúde em geral, impacto emocional nos pais e pontuação psicossocial resumida.

**Conclusões:**

A paralisia obstétrica do plexo braquial tem uma influência negativa na função do membro superior e na qualidade de vida, principalmente em relação à saúde geral, mobilidade básica, funções física e psicossocial, felicidade, dor, comportamento, saúde mental, funcionalidade do membro superior e impacto emocional nos pais.

## INTRODUCTION

Neonatal brachial plexus palsy (NBPP) could result in weakness of biceps, deltoid, and external rotators of shoulder, as well as an eventual development of contractures.^1^ The incidence of NBPP in the United States is of 1.5 per 1,000 births and in other countries, 1.3 per 1,000 births.^2^ Usually, NBPP cases resolve spontaneously, but functional deficits persist in approximately 20% of the cases. When spontaneous recovery does not occur, the upper limb has deficits, such as muscle weakness, soft tissue contractures, limited range of motion (ROM), and shortened forearm and hand regardless of motor function.^3^ ^,^ ^4^ Thus, NBPP children present reduced upper limb function compared with controls.^5^

Performing simple daily tasks can be difficult for NBPP children.^1^ Some of them undergo several types of treatment, including surgical interventions and therapeutic rehabilitation. Early home management with parent involvement is necessary to improve the upper limb function.^6^ Home-based interventions aim to increase the ROM of the affected limb and, consequently, to improve the function as well as quality of life (QOL) of this population.^7^

The World Health Organization (WHO) defines QOL as a “perception of individuals about their position in life in the context of culture and value systems in which they live, and in relation to their goals, expectations, standards, and concerns”.^8^ The diagnosis of NBPP impacts the QOL negatively.^5^ ^,^ ^9^ ^,^ ^10^ ^,^ ^11^ This is probably due to functional impairments, pain, psychosocial problems,^5^ ^,^ ^9^ ^,^ ^11^ and limitation in sports activities.^3^ ^,^ ^12^ ^,^ ^13^ Personal and environmental factors can influence the QOL of children with NBPP with regard to coping mechanisms to psychological, financial, family, therapeutic, aesthetic, and body image issues.^5^

Given the challenges faced by NBPP children, as well as relation between functional deficits of upper limb and their QOL, understanding how NBPP influences the QOL and upper limb function is essential. The combination of assessment tools for upper extremity function with QOL measures may assist practitioners with caring for whole child. Exploring these parameters may aid physical therapists in addressing functional limitations in upper extremity and QOL as it is related to the components from the International Classification of Functioning, Disability and Health Model, particularly with regard to “participation” in daily life. Furthermore, there is no publication evaluating the QOL and upper limb function in Brazilian children with NBPP. Thus, the main objective of this study was to compare the upper limb function and QOL between children with NBPP and unaffected controls.

## METHOD

This was a cross-sectional study. Children with NBPP were recruited by reviewing patients’ data records at the Medical and Statistical Archiving Service, who were cared at the Clinics Hospital of Ribeirão Preto in the period from 2002 to 2012. We obtained 47 contacts of NBPP children. The first contact was made by phone. Phone numbers of ten patients were outdated, 37 families were contacted, eight parents refused to consent to their child’s participation in the study; therefore, the article included 29 individuals. However, five were excluded due to full recovery. Twenty-four unaffected controls included normal children from a convenience sample of children taking swimming classes. Children’s parents or guardians were informed about the study objective and procedures and provided a signed written informed consent. The children also signed the consent agreement terms approved by the Research Ethics Committee of the Medical School of Ribeirão Preto of the University of São Paulo (process number 16172/2015).

Participants were divided into two groups based on their conditions: the NBPP group (NBPPG) and the unaffected control group (UCG) matched by sex and age. The UCG included 24 children (age: 10±3 years, weight: 38.0±11.1 kg, height: 1.4±0.1 m) and the NBPPG had 24 children with NBPP (age: 10±3 years, weight: 45.4±16.5 kg, height: 1.4±0.1 m), 13 had a right limb injury and 11 had a left limb NBPP diagnosis. Both groups were composed of 13 girls and 11 boys. The NBPPG were classified into the following types: upper Erb’s palsy (7), extended Erb’s palsy (10), and total palsy with no Horner syndrome (7). The injury level was verified using electroneuromyography (shown in medical records) and classified by Narakas classification.^14^

The inclusion criteria were diagnosis of NBPP and ages between five and 14 years old. The exclusion criteria were bilateral plexus lesions, complete recovery of NBPP lesions, musculoskeletal and neurologic disorders, and cognitive, auditory, and visual impairments, diagnosed by data retrieved from medical records and interviews with parents.

The upper limb function was evaluated through the Modified Mallet Scale (MMS) and Active Movement Scale (AMS). For the MMS, children were asked to perform the following movements: global abduction, external and internal rotation; hand to neck, hand to spine, and hand to mouth movements. Each movement was classified from 1 (no movement) to 5 (ROM symmetrical to unaffected side).^15^ MMS scale showed a *Kappa* value 0.78 for inter-observer reliability of individual elements and 0.76 for intra-observer reliability.^16^

Performance of the following upper limb movements was assessed using AMS: flexion, abduction, adduction, shoulder internal and external rotation, elbow flexion and extension, forearm pronation and supination, wrist flexion and extension, finger flexion and extension, thumb flexion and extension.^17^ AMS evaluated both movements without influence of gravity and movements against gravity. Each movement was scored from zero to seven (Table 1). This scale presented moderate to excellent inter-rater and intra-rater reliability for NBPP children in all evaluated movements.^16^ ^,^ ^17^ The UCG presented full active ROM against gravity, thus they received a maximum score of 7.

**Table 1:** Scores for the Active Movement Scale^17^.

Observation	Score
Gravity eliminated	
No contraction	0
Contraction, no motion	1
<50% range of motion	2
>50% range of motion	3
Full motion	4
Against gravity	
<50% range of motion	5
>50% range of motion	6
Full motion	7

The QOL was evaluated using the Pediatric Outcome Data Collection Instrument (PODCI) and Child Health Questionnaire - Parent Form 28 (CHQ). Both versions were applied and are “parent forms”, which were self-completed by the children’s parents. The evaluator instructed the parents to answer the questionnaires related to daily-life activities, covering a seven-day period for the PODCI and four weeks for the CHQ.

The PODCI has 48 items to address the child or adolescent’s overall function from parents’ perspective, under five domains: upper limb function, transfers and basic mobility, sports and physical function, comfort/pain, happiness with physical condition, and global function.^11^ This instrument has been validated for Brazilian children and adolescents with juvenile idiopathic arthritis.^18^ It is a subjective measurement tool that assesses children and adolescents aged 2-10 and 11-18 years with moderate to severe orthopedic diseases. The PODCI has four to six options per question. The final score for each domain ranges from 0 to 100: the higher, the better evaluated domain condition. The final score was obtained using Microsoft Excel™ software.^19^

Degrees of health, satisfaction, and well-being were evaluated with CHQ - 28 items, which were associated with the following domains: overall health perceptions, physical function, role/social limitations due to emotional/behavioral difficulties, role/social limitations due to physical health, body pains, behavioral/mental health, self-esteem, and change in health.^20^ Additionally, CHQ assessed the impact of child’s health on the parents’ QOL via domains related to emotions and parents’ time, family activities and cohesion.^20^ Ten concepts were then aggregated and averaged into two scores, namely psychosocial and physical summary scores. Each question presented four to six options. The score was transformed into a scale ranging from zero to 100. The higher the function, the better. A worksheet was developed for scores using Microsoft Excel™ software.^20^ This questionnaire was transculturally adapted to the Brazilian population,^21^ and presented test-retest reliability with an intraclass correlation coefficient of 0.50-0.78 for eight domains, as well as for the psychosocial summary score.^22^

All the evaluations were performed by a properly trained physiotherapist with seven years of experience in pediatric rehabilitation.

Descriptive statistical analyses of variables were performed using mean and standard deviations, as well as 95% confidence intervals of groups. Non-parametrical tests were used due to non-normally distributed data. Mann-Whitney’s tests investigated differences between groups (NBPPG and UCG) in the MMS, AMS, CHQ, and PODCI.

The effect size was classified as small, moderate, and large effects for 0.2, 0.5 and 0.8, respectively.^23^ All statistical analyses were performed using the Statistical Package for Social Sciences (SPSS), version 20.0, and the significance level was <0.05.

## RESULTS

The NBPPG has lower limb function than the UCG (Table 2). There were differences between groups in most of the PODCI domains, except for comfort/pain (Table 3). In the CHQ domains, there were differences between groups for physical functioning, body pain, behavior, mental health, change in health, emotional impact on parents, and psychosocial summary score (Table 4).

**Table 2:** Comparison of upper limb function between the groups by Active Movement Scale and Modified Mallet Scale.

	NBPPG Mean±SD	NBPPG Median	NBPPG 95%CI	UCG Mean±SD	p-value	ES
AMS						
Shoulder flexion	5.0±2.0	6.0	4.0-6.0	7.0±0.0	≤0.001	1.4
Shoulder abduction	5.0±2.0	6.0	4.0-6.0	7.0±0.0	≤0.001	1.4
Shoulder adduction	5.0±2.0	6.0	4.0-6.0	7.0±0.0	≤0.001	1.4
Internal rotation	5.0±2.0	6.0	4.0-6.0	7.0±0.0	≤0.001	1.4
External rotation	3.0±2.0	5.0	2.0-5.0	7.0±0.0	≤0.001	2.8
Elbow flexion	6.0±2.0	6.0	5.0-7.0	7.0±0.0	≤0.001	0.7
Elbow extension	6.0±2.0	6.0	5.0-6.0	7.0±0.0	≤0.001	0.7
Forearm pronation	5.0±2.0	7.0	4.0-7.0	7.0±0.0	≤0.001	1.4
Forearm supination	5.0±2.0	6.0	4.0-6.0	7.0±0.0	≤0.001	1.4
Wrist flexion	5.0±2.0	6.0	4.0-6.0	7.0±0.0	≤0.001	1.4
Wrist extension	5.0±2.0	6.0	4.0-6.0	7.0±0.0	≤0.001	1.4
Fingers flexion	6.0±1.0	7.0	6.0-7.0	7.0±0.0	0.02	1.4
Fingers extension	6.0±2.0	7.0	4.0-7.0	7.0±0.0	≤0.01	0.7
Thumb flexion	5.0±3.0	7.0	4.0-7.0	7.0±0.0	≤0.01	0.9
Thumb extension	5.0±3.0	7.0	4.0-6.0	7.0±0.0	≤0.01	0.9
MMS						
Global abduction	3.0±1.0	4.0	3.0-4.0	5.0±0.0	≤0.001	2.8
Global external rotation	2.0±1.0	2.0	2.0-3.0	5.0±0.0	≤0.001	4.2
Hand to neck	3.0±1.0	3.0	2.0-4.0	5.0±0.0	≤0.001	2.8
Hand to spine	3.0±1.0	3.0	2.0-3.0	5.0±0.0	≤0.001	2.8
Hand to mouth	3.0±1.0	4.0	3.0-4.0	5.0±0.0	≤0.001	2.8
Internal rotation	3.0±1.0	3.0	3.0-4.0	5.0±0.0	≤0.001	2.8

NBPPG: neonatal brachial plexus palsy group; UCG: unaffected control group; SD: standard deviation; 95%CI: 95% confidence interval; ES: effect size.

**Table 3:** Comparison between groups in the Pediatric Outcome Data Collection Instrument domains.

	NBPPG			Control			p-value	ES
	Mean±SD	Median	95%CI	Mean±SD	Median	95%CI		
Upper extremity	74.9±17.7	79.0	67.2-82.6	98.0±5.1	100.0	95.8-100.1	≤0.001	1.7
Mobility	95.8±5.7	97.0	93.3-98.3	99.4±1.2	100.0	98..8-99.9	≤0.001	0.9
Sports/Physical	90.4±10.2	92.0	85.9-94.8	96.7±7.0	100.0	93.7-99.6	≤0.001	0.7
Comfort/Pain	86.2±17.3	89.0	78.7-93.6	92.4±12.8	100.0	87.0-97.8	0.14	0.4
Happiness	82.6±15.4	85.0	75.9-89.2	94.6±9.0	100.0	90.8-98.4	≤0.01	0.9
Global function	86.7±7.7	88.0	83.4-90.1	96.6±4.7	98.5	94.6-98.6	≤0.001	1.5

NBPPG: neonatal brachial plexus palsy group; UCG: unaffected control group; SD: standard deviation; 95%CI: 95% confidence interval; ES: effect size.

**Table 4:** Comparison between groups in the domains of the Child Health Questionnaire.

	NBPPG			Control			p-value	ES
	Mean±SD	Median	95%CI	Mean±SD	Median	95%CI		
General health perceptions	88.1±13.0	85.0	82.6-93.6	95.0±7.2	100.0	91.9-98.0	0.04	0.6
Physical functioning	88.5±20.4	100.0	79.9-97.0	95.8±20.4	100.0	87.2-104.4	≤0.01	0.3
Role/social-emotional/behavioral	90.1±18.6	100.0	82.3-97.9	95.8±15.1	100.0	89.4-102.1	0.14	0.3
Role/social-physical	91.6±24.6	100.0	81.2-102.0	100.0±0.0	-	-	0.07	0.5
Bodily pain	81.7±17.6	80.0	74.2-89.1	91.7±11.7	100.0	86.7-96.6	0.04	0.7
Behavior	53.4±25.9	50.0	42.4-64.3	71.2±23.9	75.0	61.1-81.3	0.02	0.7
Mental health	73.8±18.3	75.0	66.1-81.5	85.8±16.4	91.8	78.9-92.7	0.01	0.7
Self esteem	81.7±19.9	87.5	73.3-90.0	87.5±22.8	100.0	77.9-97.2	0.17	0.3
Parent impact - emotional	60.4±26.3	62.0	49.3-71.5	84.9±20.5	88.0	76.3-93.7	0.001	1.0
Parent impact - time	88.9±18.1	100.0	81.3-96.6	92.3±22.5	100.0	82.8-101.8	0.20	0.2
Family activities	82.8±19.9	88.0	74.4-91.2	90.1±24.1	100.0	79.9-100.3	0.05	0.3
Family cohesion	76.0±19.4	85.0	67.8-84.2	78.3±20.0	78.3	69.8-86.8	0.63	0.1
Physical summary	54.4±9.3	57.0	50.4-58.3	58.5±4.2	59.0	56.7-60.3	0.09	0.6
Psychosocial summary	45.2±9.9	45.0	41.1-49.4	53.1±10.1	55.3	48.8-57.4	0.003	0.8

NBPPG: neonatal brachial plexus palsy group; UCG: unaffected control group; SD: standard deviation; 95%CI: 95% confidence interval; ES: effect size.

## DISCUSSION

The aim of this study was to compare the upper limb function and QOL between children with NBPP and unaffected controls. Parent’s subjective evaluation showed they believe NBPP children have worse overall function and lower QOL. This information may be useful for clinicians to notice that NBPP negatively affects the QOL of affected children in multiple aspects, based on perception of their parents, including the emotional impact on their families. Psouni et al.^24^ verified that children who suffered a NBPP are at a higher risk of using psychotropic medication in adolescence, compared to the Control Group. Furthermore, female adolescents and individuals with lower family income were at higher risk of being prescribed and using psychotropic drugs. Belfiore et al.^25^ believe that an interdisciplinary approach is necessary to determine the need for mental health referral.

Children with NBPP showed reduction in the upper limb function. Squitieri et al.^5^ applied the MMS in NBPP adolescents and Akel et al.^9^ used AMS in children, they have found similar results. According to Akel et al.,^9^ there were children with total brachial plexus and with upper trunk lesions; however, this division was not done in the present study. The NBPPG had lower scores and larger effect sizes on shoulder external rotation on both AMS and MMS, similarly to Squitieri et al.^5^ External rotation movement is widely used with other movements to perform functional tasks, such as combing the hair. Tasks involving hand’s placement on back neck also received low scores in MMS for NBPP children.

The impaired upper limb function is the domain that mostly decreases the QOL in NBPP children. The PODCI domain with the lowest score in NBPP children was upper limb function, corroborating other findings.^10^ ^,^ ^11^ ^,^ ^26^ Such result emphasizes how hard it is to manipulate objects with the upper limbs in some activities, such as lifting heavy books, pouring half gallon of milk, opening a jar that has been opened before, using cutlery, combing the hair, buttoning clothes, putting on a coat, and writing with a pencil.^19^ The effect size of this domain was the highest and reinforces the impact of NBPP on the upper limb function. In addition, the effect size of global function domain was also raised. Both domains may be related to the limited ROM in these children, which is impaired by muscle weakness, simultaneous activation of antagonists, length difference in the affected limb, and contractures.^4^ Then, NBPP children show restrictions in performance of functional tasks,^1^ which are fundamental for independence in daily-life activities and QOL,^3^ as well as for tasks that require fine motor skills (e.g. writing).^27^

Children with NBPP have low participation in sports activities. The PODCI domain of sports and physical function was reduced in the NBPPG compared with the UCG, confirming other findings.^3^ ^,^ ^10^ ^,^ ^11^ Sports and physical function domain cover tasks such as walking, running, climbing stairs, and cycling, as well as participation in non-competitive sports and games, in comparison to sports competitions with other children at the same age.^19^

In the CHQ, differences in physical functioning were observed between groups; however, the effect size was small. Physical functioning domain covered limitations related to physical functions of lower limbs, for NBPP children without limitations. The difference in the PODCI physical function domain might be related to the difficulty of NBPP children to join in activities that require the upper limb use, such as volleyball and swimming, which were some of the examples mentioned in the questionnaire. Children with NBPP have the perception that limited ROM and reduced strength affect their performance of certain movements in sports or school activities.^3^ ^,^ ^12^ ^,^ ^13^ In the current study, however, parents were the ones who showed reduced perception in the sports and physical function domain. In Kirjavainen et al.,^12^ NBPP children (n=79; 71%) had limitations in performing activities such as cycling, cross-country skiing, and swimming. These activities were questioned because they are usual to Finnish population and require bimanual function. On the other hand, Bae et al.^24^ reported that NBPP children were similar to the published normative pediatric data: 75 (88%) of NBPP children played sports, and 61 (72%) were involved in individual sports and 54 (63%) in team ones. These results showed a representative participation in several sports, including those requiring upper extremity dexterity, such as baseball, swimming, and gymnastics.^24^

Children with NBPP may be less happy than those with typical development. The NBPPG presented lower scores in the PODCI happiness domain, which addressed questions on the children’s satisfaction with their appearance, body, clothes and shoes, ability to do the same things as their peers, and overall health.^19^ Children with NBPP use compensatory patterns to perform tasks, such as opening a jar, typing, and complaining about apparent physical discrepancies when they are wearing half or quarter-length sleeves.^5^ The lowest scores in this domain may have been related to feelings of irritation, distress, and frustration, which are frequent in children, due to the differences in the level of performance of the same tasks as their regular counterparts.^9^ ^,^ ^28^ In addition, these children often feel ashamed of their appearance or size discrepancies their limbs.^3^ ^,^ ^5^

Children with NBPP have low basic mobility. Transfers and basic mobility were also reduced in NBPPG, corroborating other findings.^10^ ^,^ ^11^ In this domain, lower scores were obtained for tasks that required upper limbs use, as limited ROM affected the functionality of such children.^13^

On the parents’ perception, NBPPG children have more pain than the unaffected controls. The pain domain was assessed using the CHQ and PODCI, higher perceptions of pain were reported by parents of NBPP children in the CHQ alone. On the other hand, this phenomenon was reported by Bae et al.^10^ with the PODCI alone and by Squitieri et al.^5^ with both questionnaires. The CHQ asks about the frequency of pain/discomfort in the body for the last four weeks, whereas the PODCI is more specific, it asks if the children had pain/discomfort last week and if the pain interfered with their child’s activities and how much pain interfered in the child’s regular activities, including at home, outside home and at school. These results could be related to the numbness in the limb or pain experienced in the morning as reported by NBPP children.^13^ In addition, Akel et al.^9^ found that NBPP children with greater upper limb injury (total lesions) showed a higher perception of pain and discomfort in the affected limb than children with upper trunk lesions. However, subdivision of lesion levels was not performed in the current study.

Perception of the overall health of NBPP children’s parents is reduced. It was worse than those among parents of the unaffected controls, corroborating the findings of Akel et al.^9^ In addition, greater emotional impact was observed among parents of affected children, in agreement with previous studies on children^9^ and adolescents with NBPP.^5^ The low scores observed in these domains could be related to the concerns associated with the chronic condition. From birth, affected children often experience momentary limitations when performing daily-life activities. Furthermore, they undergo routine rehabilitation programs and surgeries.^9^ NBPP poses several challenges for children and their parents throughout their lives. Matsumoto et al.^29^ observed that parents had higher expectations from treatment than their children, however, this treatment routine often creates a sense of guilt and concern for the parents, which influences their perception of their child’s overall health, in addition to the emotional impact on their own lives.

NBPP children have low mental health in the parents’ perception. Mental health in the NBPPG was also markedly reduced compared to the UCG, corroborating other studies.^5^ ^,^ ^9^ In this domain, the CHQ included questions about feeling alone and acting in a nervous, uncomfortable, and upset manner. These behaviors could be related to feelings such as anger or irritation due to the differences in task performance when compared to that in other children.^9^ In addition, NBPP children undergo several therapies without a guarantee of functional improvement,^30^ and this uncertainty of outcomes could also trigger negative emotions, such as anxiety or fear,^13^ which could influence these results. However, it was surprise in the self-esteem domain that parents of NPBB children reported scores similar to those of unaffected controls.

The behavior of NBPP children is different than that of control children. The behavior domain was also reduced in NBPP children, corroborating with findings of Akel et al.^9^ and disagreeing with the results of Squitieri et al.^5^ This domain addresses questions about how often the child has had difficulty concentrating or paying attention and lied or cheated. This inconsistency in results present in literature may be related to each child’s personality traits.

NBPP children have lower QOL than control children of the same age and sex. Although the QOL is influenced by cultural and social aspects,^8^ in the present study, Brazilian children with NBPP presented similar results to studies conducted in other countries.^5^ ^,^ ^9^ ^,^ ^10^ ^,^ ^11^ ^,^ ^26^ The QOL involves several health components, including the ability to perform routine functional tasks, emotional well-being, and absence of pain.^29^ The evaluation of QOL in children is difficult because they are still in emotional, social, physical and cognitive development. Thus, their parents’ perspective seems to be the best measure. However, studies have shown inconsistencies in the responses about QOL from the parents and their own children’s perspective.^31^ Owing to these response inconsistencies, the QOL assessment from parents’ perspective could be considered a study limitation. As NBPP is a relatively rare condition, the small sample size can also be considered a limitation, and these results cannot be generalized for the NBPP population.

In conclusion, results showed that NBPP children presented lower upper function in the AMS and MMS when compared to unaffected controls. Parents consider NBPP as a negative influence on their children’s QOL, mainly in relation to upper limb function, overall health, basic mobility, physical function, happiness, pain, behavior, mental health, and emotional impact on their parents, as well as psychosocial summary score. Coping approaches should be included by health professionals to improve these children and their families’ QOL.
